# Peripheral Blood Mononuclear Cells and Platelets Mitochondrial Dysfunction, Oxidative Stress, and Circulating mtDNA in Cardiovascular Diseases

**DOI:** 10.3390/jcm9020311

**Published:** 2020-01-22

**Authors:** Abrar Alfatni, Marianne Riou, Anne-Laure Charles, Alain Meyer, Cindy Barnig, Emmanuel Andres, Anne Lejay, Samy Talha, Bernard Geny

**Affiliations:** 1 Unistra, Translational Medicine Federation of Strasbourg (FMTS), Faculty of Medicine, Team 3072 “Mitochondria, Oxidative Stress and Muscle Protection”, 11 rue Humann, 67000 Strasbourg, France; aaalfatni@hotmail.com (A.A.); marianne.riou@chru-strasbourg.fr (M.R.); anne.laure.charles@unistra.fr (A.-L.C.); cindy.barnig@chru-strasbourg.fr (C.B.); alain.meyer1@chru-strasbourg.fr (A.M.); anne.lejay@chru-strasbourg.fr (A.L.); samy.talha@chru-strasbourg.fr (S.T.); 2 University Hospital of Strasbourg, Physiology and Functional Exploration Service, 1 Place de l’Hôpital, 67091 Strasbourg CEDEX, France; 3 Internal Medicine, Diabete and Metabolic Diseases Service, University Hospital of Strasbourg, 1 Place de l’Hôpital, 67091 Strasbourg CEDEX, France; emmanuel.andres@chru-strasbourg.fr; 4 Vascular Surgery and Kidney Transplantation Service, University Hospital of Strasbourg, 1 Place de l’Hôpital, 67091 Strasbourg CEDEX, France

**Keywords:** cardiovascular diseases, mitochondrial dysfunction, circulating cells, PBMCS, platelets, oxidative stress, reactive oxygen species (ROS), mitochondrial DNA (mtDNA), biomarkers, herat failure

## Abstract

Cardiovascular diseases (CVDs) are devastating disorders and the leading cause of mortality worldwide. The pathophysiology of cardiovascular diseases is complex and multifactorial and, in the past years, mitochondrial dysfunction and excessive production of reactive oxygen species (ROS) have gained growing attention. Indeed, CVDs can be considered as a systemic alteration, and understanding the eventual implication of circulating blood cells peripheral blood mononuclear cells (PBMCs) and or platelets, and particularly their mitochondrial function, ROS production, and mitochondrial DNA (mtDNA) releases in patients with cardiac impairments, appears worthwhile. Interestingly, reports consistently demonstrate a reduced mitochondrial respiratory chain oxidative capacity related to the degree of CVD severity and to an increased ROS production by PBMCs. Further, circulating mtDNA level was generally modified in such patients. These data are critical steps in term of cardiac disease comprehension and further studies are warranted to challenge the possible adjunct of PBMCs’ and platelets’ mitochondrial dysfunction, oxidative stress, and circulating mtDNA as biomarkers of CVD diagnosis and prognosis. This new approach might also allow further interesting therapeutic developments.

## 1. Introduction

Cardiovascular diseases (CVDs) rank as one of the first diseases leading to death worldwide [1,2]. The 2019 report of the American Heart Association shows that between 2013 and 2016, CVDs, including hypertension, heart failure (HF), coronary heart disease, and stroke, were present in about 48% of patients older than 20 years in the United States [3,4,5]. Significant progress has been made concerning CVD diagnosis and therapies, particularly considering neuro-hormonal modulation, such as natriuretic peptide (NP)-guided therapy [2,6,7,8,9], but it seems that a plateau has begun to be reached, suggesting new approaches. In this view, since CVDs are generally systemic diseases, an attempt based on circulating cells might be proposed to better understand CVD pathophysiology and to discover new biomarkers. Indeed, growing evidence suggests that the assessment of mitochondrial respiratory function of circulating peripheral blood mononuclear cells (PBMCs) and platelets might be viewed as a marker to detect the mitochondrial dysfunction in different tissues, including the heart [10,11,12,13,14].

The myocardium possesses one of the highest number of mitochondria in the body, allowing heart pumping activity through ATP production. Mitochondria are known contributors to the pathogenesis and outcome of several cardiovascular diseases. Indeed, regardless of cardiac disease etiology, most evidence demonstrates that mitochondrial dysfunction is widely observed in the pathological heart.

Mitochondrial dysfunction might be inferred from tissues’ or cells’ oxygen consumption (reflecting mitochondrial oxidative capacity) and the mitochondrial membrane potential (reflecting the ability of the electron transport system to maintain the gradient of proton driving ATP production). Thus, the failure of mitochondria to produce ATP results in an energy deficit, impairing cells, and finally, organ function [14,15,16,17,18,19].

Mitochondria have also been identified as significant sources of reactive oxygen species (ROS) [20]. Research reveals that oxidative stress, due to increased ROS and/or reduced antioxidant capacity, plays a considerable role in the development of HF and determines patient prognosis. Increased ROS accumulation and inflammation play a key role in the cardiac and vascular functional and structural damage underlying all major causes of CVDs [21]. However, the fundamental mechanism of ROS production in HF deserves to be further investigated [22]. Thus, it would be interesting to further monitor ROS levels and mitochondrial function in circulating cells in order to improve both diagnosis and follow-up of patients with CVDs.

Indeed, if tissue biopsies are relevant to the investigation of pathological changes and study of mitochondrial function in diseased organs, they are invasive and not always feasible. Alternatively, peripheral blood mononuclear cells (PBMCs) and/or platelets represent an easily available population of cells allowing mitochondrial function studies. Analysis of the energetic profile (mitochondrial function) of circulating blood cells in experimental animals and humans appears as a new research field with potential applications in the development of disease biomarkers in several settings, including respiratory and CVDs [10,11,12,13,14,19,23,24].

This review presents data exploring the PBMCs’ and platelets’ mitochondrial function, together with their ROS production and mitochondrial DNA release in order to assess whether such key parameters are modified and might be considered as biological markers of CVDs with diagnosis, prognosis, and even prognosis interests.

## 2. Is Mitochondrial Function Accessible in all Circulating Cells in the Blood?

### 2.1. Classification of Circulating Cells

**Figure 1:**
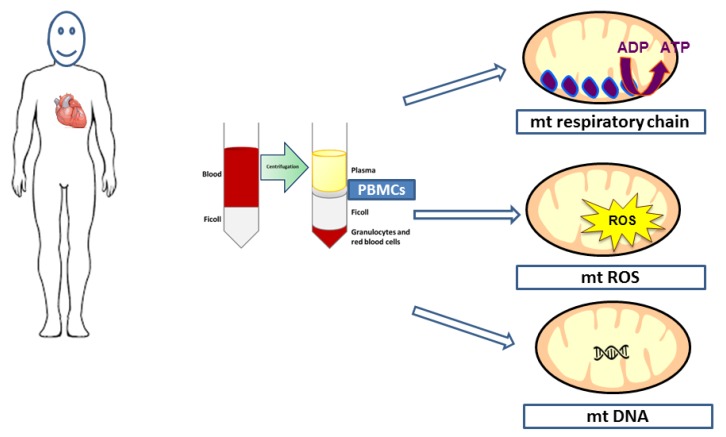
Density gradient centrifugation of whole blood allows peripheral blood mononuclear cells (PBMCs) isolation and then mitochondrial respiratory chain, reactive oxygen species, and DNA analysis.

There are many circulating cells in the blood, ranging from different population subtypes of white cells involved in immunity and inflammation, to platelets modulating blood aggregation and to red cells mainly transporting O_2_. Several techniques might be used to separate blood cells in the blood, but gradient centrifugation is generally performed (Figure 1). Theoretically, the oxidative capacity of all circulating cells—through mitochondrial respiratory chain complexes activities assessment—might be explored, but there is a noticeable exception. Unlike in birds, for instance, human red blood cells do not present with mitochondria [25].

The PBMC fraction consists of lymphocytes (T, B, and natural killer cells), monocytes, and dendritic cells. Circulating lymphocytes represent a mixed population of cells and ensure cellular or humoral immunity. Many types of lymphocytes can be distinguished: B cells produce antibodies, T cells support cellular immunity, and natural killer cells have their own cytolytic activity. The production of monoclonal antibodies specific for an expressed antigen can be conducted for immunophenotypic lymphocyte classification: Cluster of differentiation (CD) was created to group antibodies that recognize the same antigens. However, to date, no clear data are available on these specific cells’ subtypes concerning their mitochondrial respiration. Monocytes, with a uni-lobular nucleus, have an important role in phagocytosis and the innate immune system.

Platelets are un-nucleated cells produced by cytoplasm fragmentation of megakaryocytes in the bone marrow, circulating in the peripheral blood for 7 to 10 days. They play a significant role in homeostasis and are essential for thrombus formation during the hemostatic process and are largely involved in thrombosis, myocardial infarction, stroke, and phlebitis. Platelets thus play an important role in CVDs, both in the pathogenesis of atherosclerosis and in the development of thrombotic events when presenting with qualitative and/or quantitative impairments [11,13,26,27]. Circulating platelets possess numerous mitochondria, can be obtained easily even from critically ill patients, and their isolation is performed routinely with success [28].

### 2.2. Isolation and Mitochondrial Respiratory Chain Activities’ Determination in PBMCs and Platelets

Mitochondria are the main source of cellular energy, coupling the oxidation of fatty acids and pyruvate to the production of high amounts of ATP through the mitochondrial electron transport chain (ETC) [29]. Briefly, free electrons are transferred from complex I to complexes II, III, and IV of the ETC, thereby allowing complexes I, III, and IV to extrude protons from the matrix. The return of H+ ions from the mitochondrial membrane interspace towards the matrix allows the complex V to phosphorylate ADP into ATP.

The function of each complex is investigated by using a spectrophotometer and can be performed in cellular or tissue samples. Besides the determination of oxygen consumption, ATP synthesis and the mitochondrial membrane potential can also be investigated [30]. Specifically, Hsiao and Hoppel presented an optimal comprehensive method for analyzing the ETC activity in PBMCs [31]. There are two general techniques that have been used in vitro for the assessment of mitochondrial function and detection of delicate changes in the respiration rate of mitochondria in PBMCs and platelets isolated from peripheral blood by measuring oxygen consumption [12,13,32]. The first method is the extracellular flux analyzer. This technique provides efficient, comprehensive, and highly reproducible results and is commonly used to measure cellular bioenergetics function in intact and permeabilized cells [33]. A distinct trait of this protocol compared to others is that it does not entail mitochondrial isolation and can be operated using a minimal number of cells [33]. The second method is high-resolution respirometry (Oroboros O2K), which permits active investigation of metabolic pathways [12] and requires the availability of sufficient numbers of cells [10].

Although circulating platelets count for small numbers of functional mitochondria, they have high energy consumption levels and have been used widely to study the mitochondrial function in human disease due to their accessibility [34]. This is confirmed by a review by Kramer et al. presenting the maximal mitochondrial oxygen consumption devoted to the bioenergetic function in circulating platelets, monocytes, and lymphocytes. Interestingly, there is a distinct metabolism program between circulating platelets and leukocytes that could act as different sensors of the metabolic and inflammatory stress in many diseases [13].

## 3. Mitochondrial Respiratory Chain Complex Activities of PBMCs and Platelets in Patients with Cardiovascular Diseases

### 3.1. PBMCs Mitochondrial Respiratory Chain Activity in Cardiovascular Diseases

Interestingly, when evaluating mitochondrial respiratory chain complexes’ activity in PBMCs in heart failure patients, Li et al. demonstrated that mitochondrial oxygen consumption, particularly in complex I and II, was significantly smaller as compared to the control group [29]. Such depressed PBMC mitochondrial function was observed in patients with early-stage congestive heart failure (CHF, asymptomatic patients) [29]. Possible explanations of this reduction in the electron transport chain activity in PBMCs are increased mitochondrial mitophagy and decreased biogenesis per mononuclear cell [29]. Moreover, the mitochondrial respiration was inversely related with inflammatory factors, such as high sensitivity C-reactive protein, IL6, and TNF-α. Thus, impaired mitochondrial respiratory functions of PBMCs characterize heart failure patients. Accordingly, a significant reduction of NDUFC2 expression, a subunit of mitochondrial complex I, has been detected in peripheral circulating mononuclear cells in patients with acute coronary syndrome [35]

More generally, there are several factors that might disrupt the function of the circulating leukocyte mitochondrial respiratory chain in CHF. Increased intracellular oxidants could induce mitochondrial permeability transition and inhibit respiratory coupling, which reflects mitochondrial respiratory chain disruption [36,37]. Kong et al. observed a reduction in the leukocyte, lymphocyte, and monocyte mitochondrial transmembrane potential (MTP) in congestive heart patients, in association with apoptosis and increased inflammation and ROS formation [37]. This decrease was more notable in the edematous CHF group when considering lymphocytes. Additionally, increased ROS led to mitochondrial depolarization [37]. Further, the percentage of apoptotic cells was greater in PMN than PBMCs (42.9% vs. 20%, respectively).

Song et al. found lower MTP and higher ROS levels in lymphocytes of CHF patients at low risk associated with increased serum NT-ProBNP, a diagnosis and prognosis biomarker in heart failure [36]. Furthermore, Coluccia et al., analyzing the mitochondrial membrane potential by cytofluorometric TMRM and JC-1 staining, found significant mitochondrial depolarization in PBMCs among HF patients after the administration of inflammatory stimulus lipopolysaccharide (LPS) [38]. The ultrastructural changes in mitochondria PBMCs showed a decrease in the index associated with the loss of inner mitochondrial membrane (IMM) and with an increase in the percentage of the apoptotic cells and mitophagy in HF-PBMC individuals, both at baseline and after LPS stimulation. The impairment of the inner mitochondrial membrane in PBMCs might reflect the impairment of the electron transport chain mitochondrial uncoupling [38] (Table 1). Thus, PBMCs’ mitochondrial respiration can be considered as an innovative model to investigate the pathophysiology of CVDs.

### 3.2. Platelets’ Mitochondrial Respiratory Chain Activity in Cardiovascular Diseases

Circulating platelets contain small number of functional mitochondria (averaging four mitochondria/platelet), but they are very metabolically active with a high rate of ATP turnover [39]. Platelets have higher oxygen consumption rates compared to leucocytes, since higher levels of ATP are required for the normal functioning of ion channels that maintain the intracellular ionic balance, essential for preventing platelet activation in basal conditions [13]. In platelets, mitochondrial complex III and IV are very low, underlying the severe impact of mitochondrial damage on platelet function. Electron transport chain activity in platelets is altered in many diseases [34]. However, there are few data in CVDs.

In resting platelets, mitochondrial respiration accounts for three-quarters of the energy production, with glycolysis providing the remaining [40]. The metabolic pool of ATP and ADP is located in the cytoplasm whereas non metabolic ATP and ADP are segregated into dense (δ) granules (storage pool); they are secreted during cellular stimulation and are essential for the late phase of aggregation [41]. Another important platelet trait is the fact that mitochondrial complex III and IV proteins are few, leading to increased sensitivity toward mitochondrial dysfunction [13]. Many studies have demonstrated an interest in monitoring platelet mitochondrial respiration in diabetes, Alzheimer’s, or Parkinson’s disease [11]. Following platelet activation, mitochondrial respiration and glycolysis enhance extra metabolic ATP production, thus sustaining shape change, aggregation, and secretion [11,39,42]. Such increased energy consumption is a main determinant of platelet function.

**Figure 2:**
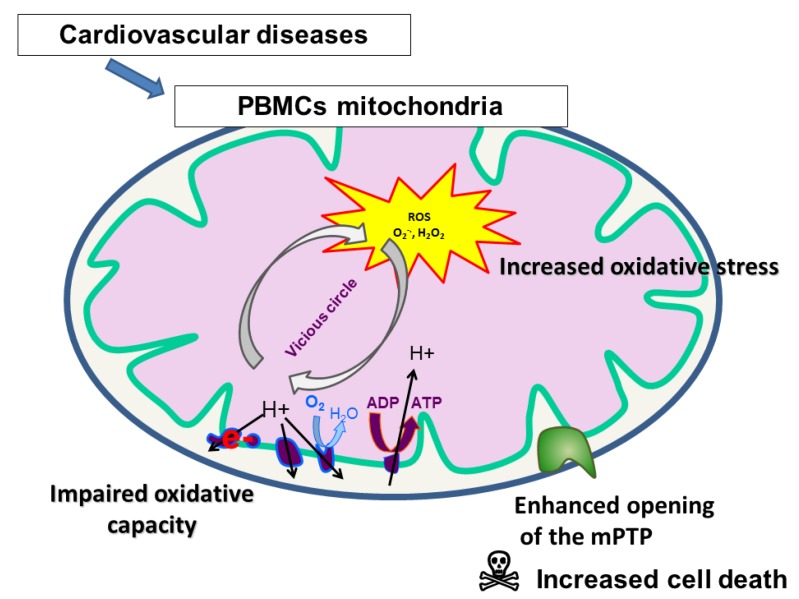
Mitochondrial alterations in PBMCs or platelets during cardiovascular diseases.

In cardiogenic shock (when the trigger is hypoperfusion), there is inhibition of platelet mitochondrial respiratory chain enzymes similar to that observed in sepsis. According to some authors, salicylic acid or its derivatives may interfere with platelet mitochondrial function, mainly acting as uncoupling agents. However, this issue still deserves further studies [42,43,44]. Petrus et al. shed light on the association between hyperpolarization of the mitochondrial membrane, ROS formation, and platelet secretion, and, for instance, diabetic patients had a lower platelet oxygen consumption rate associated with increased ROS generation [11,12]. Furthermore, circulating platelet mitochondria are not restricted to the generation of ATP, but also have an important role in initiating platelet activation through many interlinked mitochondrial processes [11,34]. Impairment of the electron transport chain leads to increased generation of ROS, which triggers platelet activation and, potentially, to a reduced mitochondrial membrane potential and mitochondrial permeability transition pore opening (Figure 2) [11].

On the other hand, Nguyen et al. recently observed that the platelets of patients with pulmonary hypertension secondary to left heart diseases demonstrated an enhanced maximal respiratory capacity despite a normal basal oxygen consumption rate [45]. Increased fatty acid oxidation, together with the metabolic syndrome, likely contributed to this result. Further and interestingly, platelets’ bioenergetics correlated with right ventricular dysfunction but not clearly with hemodynamic in these group 2 pulmonary hypertension (PH) patients, suggesting that non-hemodynamic parameters might play a significant role in such a setting.

## 4. Mitochondrial ROS Production and Antioxidant Defense of PBMCs and Platelets in Patients with Cardiovascular Diseases

### 4.1. Measurements of ROS in Circulating Cells

ROS include superoxide, H_2_O_2_, and peroxynitrite, thought to be the most common and important biological oxidants. In the cardiovascular system, different sources of ROS coexist, and NADPH oxidase, xanthine oxidase, and uncoupled eNOS, together with mitochondrial ROS, participate in endothelial dysfunction in relation to inflammation, leading to a worse prognosis. ROS production results from enzymatic reactions in different cell components, including mitochondria, and, is associated with normal basal metabolic energy generation. In the mitochondria, ROS are physiologically produced mainly across mitochondrial complex I and III of the ETC [22,49,50,51,52,53]. Thus, a normal balance of ROS is essential for cellular functions; however, once the level of ROS surpasses the standard concentration, cellular damage will result, leading finally to apoptosis and cellular death [54,55]. Therefore, an accurate and potent detection method of ROS is crucial for cardiovascular system studies [56], but ROS measurement with high accuracy is still challenging because of ROS’ short half-life [57]. Griendling et al. listed all measurement approaches of ROS in detail [58]. In a biological system, the gold standard for measuring ROS in the form of free radicals is thought to be EPR (electron paramagnetic resonance), also recognized as electron spin resonance [55,57,58]. Other measuring techniques of ROS include chemical assays for superoxide anion radicals (O_2_^−^), hydrogen peroxide (H_2_O_2_), or peroxynitrite (ONOO^−^) with fluorescence analysis in the presence of redox sensitive probes or direct chemiluminescent assays [58].

Another frequent method used in clinical setting to measure ROS is the measure of byproducts, such as lipid peroxidation through malondialdehyde (MDA), 4-Hydroxy-Trans-2-Nonenal (HNE), and isoprostanes F_2_-IsoPs determination [57,58]. Additionally, oxidative modification of protein and nucleic acid is a classic approach in cardiovascular cells [57,58]. For example, ELISA (enzyme-linked immunosorbent assay) has been recognized as the most common measuring technique used [57]. On the other hand, flow cytometry is the most powerful technique for single cell analysis of the immune system, in particular for leukocytes and platelets [55]. Many fluorescent probes are used for ROS detection in blood cells via flow cytometry [55]. For illustration, DCFH-DA, DAF-2 DA/DAF-FM DA, DHR123, and DHE are all intercellular probes and are detected as green fluorescence except DHE, which is detected as red fluorescence for both leukocytes and platelets. However, there are multiple artifacts related to the DCFH-DA probe and its use remains discussed [59]. Thus, although progress is still to be performed for oxidative stress evaluation, PBMCs can be incubated with chemiluminescent, bioluminescent, or fluorescent redox active probes to detect cytoplasmic or mitochondrial ROS. Particularly, mitochondrial ROS evaluation is possible with specific probes that can pass through the mitochondrial membrane by the addition of a triphenylphosphonium group to a fluorescent probe, like mitosox, which is an analogue of DHE, and for selective detection of H_2_O_2_ within the mitochondria, MitoPY1 can be used with imaging techniques [60].

Further, the quantitation of reactive species metabolites, ROS scavengers, and antioxidant enzymes can be obtained from chromogenic and enzymatic assays from culture supernatants. Gene expression analysis of PMBCs also allows assessment of antioxidant systems and of other molecules modulating intracellular oxidative stress, such as the *OXPHOS* genes. Finally, quantitative assessment of the mitochondrial structure and function provide additional information when oxidative stress has mitochondrial genesis.

### 4.2. Mitochondrial ROS in PBMCs in CVDs

#### 4.2.1. Mitochondrial ROS in PBMCs in Heart Failure

Oxidative stress plays a key role in the development and progression of CVDs and could be used as an indirect marker to predict disease severity and prognosis [61,62,63]. In this context, mitochondrial dysfunction appears to have increased importance [17,64]. Indeed, high levels of ROS and increased production of superoxide anion by neutrophils have been observed in the blood of HF patients, and white blood cells and platelets producing ROS can amplify oxidative stress and organ damage in HF [48,65]. A recent study showed that circulating PBMCs present structural and functional derangements of mitochondria with overproduction of ROS in HF [38]. Besides, a significant reduction of respiration was associated with a higher mitochondrial ROS production in PBMCs of patients with moderate to severe CHF compared to mild CHF [22]. Furthermore, there was a positive correlation between mitochondrial ROS formation and oxidative DNA damage and plasma BNP levels, which are related to the severity of HF. In CVDs, lymphocytes and monocytes play a key role in atherogenesis, modulating the inflammatory and immune response. Indeed, PBMCs would undergo changes similar to failing cardiomyocytes in HF [36]. Based on these data, the use of circulating leukocytes may become a relevant biomarker in cardiovascular diseases and might serve to better understand its pathogenesis [66].

The mechanisms by which mitochondrial ROS in PBMCs are increased in CVDs are multifactorial. Enhancement of myocardial ROS might stimulate ROS generation in PBMC mitochondria via the mechanism of ROS-induced ROS generation upon the passage of circulating PBMCs through the heart. Indeed, the proportion of mitochondrial ROS-loaded blood cells is higher in the coronary sinus than in the peripheral veins of CHF patients [48]. Another hypothesis is the role of inflammatory factors present in HF, such as circulating cytokines, that trigger ROS generation [29]. Further, in heart failure, tissue hypoxia may trigger an increase in the production of ROS, which is a strong stimulus of pro-inflammatory cytokines, such as IL6 and TNF-α [67]. Li et al. confirmed the involvement of mitochondrial dysfunction of PBMCs in the pathophysiology of heart failure; extreme inflammation and decreased antioxidant capacity were closely associated with heart diseases, especially in early stage heart failure patients [29].

Other markers of oxidative stress have been described, such as myeloperoxidase (MPO), oxidized low density lipoproteins (oxLDL), and F_2_Isoprostane [66]. Elevated lipid peroxidation has been shown to be associated with the severity of HF, such as malondialdehyde (MDA) and 4-Hydroxy-2-nonenal (HNE) [68]. In addition, two studies showed a positive correlation between the total plasma peroxide levels (reflecting oxidative stress index) in leukocytes with serum NT-proBNP [8,36]. Mondal et al. demonstrated that HF patients with implanted left ventricular assist devices exhibit excessive production of ROS as well as DNA damage in circulating leukocytes [47]. Similarly, Garcia Anastacia et al. observed increased ROS level and deteriorated mitochondrial respiratory capacity in circulation PBMCs in pediatric HF patients who underwent cardiac transplant [46]. 

#### 4.2.2. Mitochondrial ROS in Arterial Hypertension, Coronary Artery Disease, and Stroke

Yasunari et al. measured the oxidative stress of circulating leukocytes in both hypertensive and diabetic patients and concluded that the level of oxidative stress was significantly increased in arterial hypertension [69]. This study used peripheral leukocytes as a biomarker to detect hypertension-related vascular damage [51]. In fact, the role of measuring ROS in leukocytes in hypertensive patients might help monitor the effect of treatments [51].

In PBMCs, the evaluation of oxidative stress and mitochondrial function in coronary artery disease has been attempted via assessment of the gene expression profile of complex I subunit (NDUFc2). Raffa et al. found a significant reduction of complex I subunit with increased levels of ROS and decreased ATP levels [35].

Only a few works in the literature have demonstrated the role of ROS in circulating cells in the development of stroke [51]. Aizawa et al. showed that in stroke patients, the ROS levels of peripheral mononuclear cells (circulating neutrophils) are increased compared to controls [70].

### 4.3. Mitochondrial ROS in Circulating Platelets in CVDs

It is now clear that mitochondria modulate the pro-thrombotic function of platelets through energy generation, redox signaling, and apoptosis initiation [71,72,73]. Thus, studies have related increased platelet activation with mitochondrial hyperpolarization and ROS production. Yamagishi et al. demonstrated that hyperglycemia induces hyperpolarization in normal platelets, resulting in the production of mitochondrial ROS and subsequent activation [74]. Furthermore, Avila et al. observed in diabetic patients that platelets had decreased rates of oxygen consumption and hallmark signs of increased ROS production [75]. Preserving platelet mitochondrial function may therefore allow a decrease of the risk of thrombotic events in diabetic patients [76].

## 5. Circulating Mitochondrial DNA (mtDNA) Originating from PBMCs and Platelets in Patients with Cardiovascular Diseases

### 5.1. Circulating Mitochondrial DNA (mtDNA) Originating from PBMCs in Patients with Cardiovascular Diseases

Adequate numbers of mtDNA (free-cell mtDNA) or (circulating mtDNA) are important for mitochondrial as well as cellular function. mtDNA are released by cells undergoing stress or having pathological events [77]. MtDNA encodes 2 ribosomal RNAs, 22 transfer RNAs, and 13 polypeptides of the respiratory chain [78]. Mitochondria contain several copies of mtDNA. The number of mtDNA copies in cells correlates with the size and number of mitochondria, which change under different energy demands and oxidative stress and under different pathological conditions. The mtDNA copy number or content reflects the mitochondrial function through the mitochondrial enzyme activity and ATP production [79]. Quantification of the mtDNA copy number of PBMCs using real-time polymerase chain reaction (PCR) was found to produce consistent and reproducible results [80].

Unlike nuclear DNA, mtDNA is vulnerable to ROS damage because of the lack of histone protection and effective DNA repair mechanisms. When mtDNA damage occurs, it results in mitochondrial dysfunction, inflammation, and cell senescence participating in the pathogenesis of CVDs and atherosclerosis. The mtDNA copy number might reflect the level of mtDNA damage, potentially being a biomarker of mitochondrial function and a predictor of CVDs’ risk and prognosis [77,79,81].

Studies have tested mtDNA for the evaluation of CVDs [81,82]. High levels of circulating mtDNA behave as a danger-associated molecular pattern molecule (DAMP), enhancing inflammation and organ damage [83]. In addition, the effective release of mtDNA requires antigen-presenting cells, such as mononuclear and lymphocytes cells, to be involved [84]. Bliksøen et al. observed a correlation between increased mtDNA content and the incidence of myocardial infarction, suggesting mtDNA as a diagnostic biomarker for acute myocardial infarction (AMI) [83]. Likewise, previous evidence emphasized that mtDNA damage might promote atherosclerosis through mitochondrial impairment [85]. As an illustration, Fetterman et al. studied mtDNA damage in PBMCs in patients presenting with diabetes mellitus, clinical atherosclerosis, and CVDs through the isolation of lymphocytes and monocytes. They found that mitochondrial DNA impairment was directly related to oxidative phosphorylation impairment, which ends up with oxidative stress and organ dysfunction [86]. However, in this study, the author found no changes in the mtDNA copy number between the three groups. Sudakov et al., indicated an increase in the circulating mtDNA content in the blood of patients with acute coronary syndrome, which could be a biomarker for the probability of death from myocardial infarction [87].

Studies support the notion that a lower level of mtDNA content indicates a high risk for CVD and sudden cardiac death [81] but others suggested that increased circulating mtDNA content was linked with reduced LV diameters and volumes and thus enhanced cardiac function [77]. By the way, at least, peripheral blood mtDNA might be a predictor of heart characteristics. Chen et al. performed a study to reveal the association between the peripheral mtDNA copy number in leukocytes and risk of CHD. A correlation between the circulating mtDNA content and the formation of atherosclerotic plaque suggested a connection among low mtDNA and a high risk of coronary heart disease [88]. Huang et al. conducted studies in heart failure and acute myocardial infarction patients with consistent results. Both patients type showed lower mtDNA content than the control group [89,90]. Discrepancies in these results might be related to the disease severity, aging, or other risk factors factor that may modify directly or indirectly the outcome. Also, the site of mtDNA extraction might be important. Indeed, in one study, the mtDNA was extracted from platelet-poor plasma while other studies have investigated mitochondria from leukocytes.

Taken together, although still needing further analysis, decreased circulating mtDNA might potentially be assumed to be a risk factor for heart failure and used as a biomarker for cardiovascular disease prognosis.

Similarly, in ischemic stroke patients, Lien et al. quantified the mtDNA content in peripheral leukocytes and found a significant reduction compared to the control individuals [91]. Furthermore, Zhang et al., in patients at risk for atherosclerosis, observed an inverse correlation between the mitochondrial copy number and the risk of sudden cardiac death [92] (Table 2).

### 5.2. Circulating Mitochondrial DNA (mtDNA) Originating from Platelets in Patients with Cardiovascular Diseases

Several physiological stimuli that cause platelet activation at low concentrations could induce platelet apoptosis at higher concentrations. This type of dual signaling is potentially important in the regulation of coagulation. Increased platelet apoptosis has been reported in a number of pathologies, including type 2 diabetes [93]. Activated platelets can release functional mitochondria and mtDNA. Beyond the measurement of mitochondrial function in patients with disease, and due to its lack of a nucleus, platelets provide a unique source of mtDNA [71]. An increasing number of studies support the idea that evaluation of the bioenergetic function in circulating platelets may represent a peripheral signature of mitochondrial dysfunction in metabolically active tissues (brain, heart, liver, skeletal muscle). Indeed, owing to their easy accessibility, there is interest in the use of platelets to study mitochondrial (dys) function in human disease over time. Accordingly, impairment of mitochondrial respiration in peripheral platelets might have potential clinical applicability as a diagnostic and prognostic tool as well as a potential biomarker in treatment monitoring. In sepsis, an alteration in the bioenergetics of platelet mitochondria was directly correlated with the clinical outcome [94].

In CVDs, there are few studies on circulating platelets’ mitochondrial dysfunction. Baccarelli et al. suggested that platelet mDNA methylation may be implicated in the etiology of CVDs [95]. Regarding the fact that cardiovascular diseases are strongly influenced by platelet function though acute thrombotic and atherogenic mechanisms, we can expect that evaluation of the bioenergetic function in circulating platelets may represent a potential biomarker of CVD susceptibility, prognosis, or treatment.

An experimental evaluation of atherosclerosis by Yu and co-workers displayed that mtDNA damage was recognized in circulating monocytes, as well as decreased complex I and IV, which were associated with mitochondrial dysfunction [96]. However, this study showed an independent relation between atherosclerosis and reactive oxygen species, as it showed that those at high risk of atherosclerosis have extensive mtDNA damage with no increase in ROS levels [96].

## 6. Conclusions

In summary, this review outlines the importance of mitochondrial function in circulating blood cells and particularly, its relationship with CVDs. Impaired mitochondrial respiratory chain activity and ATP generation, changes in mitochondrial DNA content, and increased ROS formation in PBMCs and likely in platelets are often associated with several types of cardiovascular diseases. Currently, an evaluation of the mitochondrial function of circulating cells in human blood for cardiovascular disease might be considered as a new noninvasive approach that deserves further studies to improve its diagnosis and prognosis interest. Also, mitochondrial function and ROS and mtDNA involvement in CVD physiopathology support that a better knowledge of these aspects might open new therapeutic perspectives.

## Figures and Tables

**Table 1:** Mitochondrial function, oxidative stress, and apoptosis in circulating blood cells during cardiovascular disease.

Population Characteristics	Study Design/ Cells Analyzed	Mitochondrial Function	Oxidative Stress ROS Production/ Antioxidant Level	Cell Viability/Apoptosis	Results	References
HF pediatric patients with single ventricle (SV) congenital heart disease	PBMCs	-Oxygen consumption rate (Seahorse) -Mitochondrial respiration (oroboros)	ROS (Amplex red dye)	NA	-Respiratory capacity, coupling efficiency and mitochondrial oxygen flux were reduced in SV patients. -ROS was higher in SV patients	Garcia Anastacia et al., 2019, Circulation (Abstract) [46].
-Mild Congestive Heart Failure patient (CHF) (Class I-II) *n* = 15, 14 male, 1 female Age: 63 ± 13 yo EF: 44.3 ± 14.5 % -Moderate-to-severe CHF (Class III) *n* = 16, 15 male, 1 female Age: 61 ± 14 yo EF: 26.9 ± 6%	PBMCs	-Mitochondrial respiration (oroboros) -Maximal electron transfer system capacity (ETS)	Assessment of ROS generation in permeabilized PBMCs before and after addition of mitochondrial oxidative phosphorylation uncoupler (FCCP) urinary 8-OHdG, a biomarker of oxidative DNA damage	N/A	Mitochondrial respiratory capacity of class III HF was lower than class II patients. -ETS capacity was significantly reduced in class III compared to class I–II -Mitochondrial ROS level was higher in class III CHF compared to class I–II patients, before and after FCCP.	Shirakawa et al., 2019, Scientific Report. [22]
Chronic HF patients *n* = 15, 12 male, 3 female Age: 56.6 ± 10.8 yo EF: 28 ± 8% Control group *n* = 10, 8 male, 2 female Age: 49.3 ± 8 yo EF: 65 ± 2%	PBMCs Basal and modulation by LPS	Mitochondrial membrane potential (TMRM and JC-1 staining).	-For cytoplasmic oxidative stress evaluation: PBMCs were incubate with 5 µM 2′,7′-dichlorofluorescein diacetate at 37 °C for 10 min. -For mitochondrial oxidative stress evaluation: (MitoSOX™ Red mitochondrial superoxide) -For antioxidant system (SOD GPx levels)	Assessment of overall cell damage Mitochondrial area percentage of intact cristae, and loss of inner mitochondrial membrane (IMM) -Cell damage (Annexin-v and P1 staining by flow cytometric analysis) -Assessment of mitophagy flux (gene expression by RT-PCR quantitation).	**Baseline** -Cytoplasmic ROS: no difference between HF-PBMCs and healthy subject. -Mitochondrial ROS: increased in HF-PBMCs as compared to controls -Index associated with the loss of inner mitochondrial membrane was lower in HF patients -mitophagy flux: increased autophagy genes in HF-PBMCs **After LPS** -Mitochondrial membrane potential: depolarization in PBMCs of HF patients (*p* < 0.05). -Antioxidant system: reduced SOD (*P* < 0.05 and <0.01) and GPx (*p* < 0.05) activity in HF-PBMCs -Cytoplasmic ROS: HF-PBMCs shows marked increase cytoplasmic ROS than control group. (*p* < 0.05) -Mitochondrial ROS: increased in HF patients (*p* < 0.05). - Index associated with the loss of inner mitochondrial membrane was more deteriorated after stimulation, and reduction of mitochondrial area with intact cristae in HF-PBMCs than in healthy group (*p* < 0.01) -Cell damage: apoptotic cell percentage was increased in HF patients. (*p* < 0.05) -Mitophagy flux: the response in HF-PBMCs was increased much more after stimulation.	Coluccia et al., 2018, Oncotarget. [38]
Congestive heart patients (CHF) *n* = 20, 16 male, 4 female Age: 68.9 ± 8 yo EF: 24.9 ± 5.9% -Control group *n* = 15, 13 male, 2 female Age:63.3 ± 9.4 yo EF: 60.0 ± 5.3%	Leukocyte were isolated by gradient centrifugation to measure cellular lipid, protein, PARP & AIF Modulation: Activation of PARP	N/A	C-reactive protein, N-terminal probrain-type natriuretic peptide, oxidative nitrative stress, plasma total peroxide level (PRX), total plasma antioxidant capacity (TAC)and oxidative stress index (OSI), Leukocyte lipid peroxidation, and protein tyrosine nitration (NT)were evaluated. PRX was determined by Oxystat and TAC was detected by OxiSelect™ TAC Assay kit	poly (ADP-ribose) polymerase (PARP), and apoptosis inducing factor (AIF) was measured	In CHF patients, plasma PRX level was markedly increased suggesting the increase of oxidative stress in this group. Oxidative stress of leucocytes increased in CHF group. PARP activity and AIF in circulating mononuclear cells of CHF group was higher than in the control group. A positive correlation was demonstrated between oxidative stress (Plasma PRX level, OSI) and PARP activation in circulating leukocytes with pro-BNP levels of CHF.	Bárány et al., 2017 Oxidative Medicine and Cellular Longevity. [8]
Pulmonary hypertension patients (PH group classified as WHO Group 2) *n* = 20, 10 male, 10 female Age: 69 ± 7.4 Control group *n* = 20, 10 male, 10 female Age: 69.4 ± 17.6	Platelets	Oxygen consumption (Seahorse) Extracellular acidification rate (Seahorse)	ROS level analyzed using MitoSOX	N/A	Maximal oxygen consumption rate was significantly increased compared to controls Activity of complex II tended to increase in Group 2 PH platelets compared to controls (*p* = 0.09). Enhanced maximal capacity correlates negatively with right ventricular stroke work index No change with administration of inhaled nitrite, a modulator of pulmonary hemodynamics.	Nguyen et al., 2019, Plos one. [45]
CHF*n* = 54, male Age: 60 ± 10 EF% 33.3 ± 7.7 Control group *n* = 30, male Age: 61 ± 10 EF% 65.1 ± 7.3	PMBCs (peripheral blood Lymphocyte Serum NT-ProBNP level were assessed	Mitochondrial transmembrane potential (MTP) Analyzed by flow cytometry described as JC-1 fluorescence ratio	ROS level of PBMCs were investigated. Described as DCF fluorescence intensity.		CHF patients experienced decreased MTP, (and increase level of ROS of lymphocytes (intensity 11.12) than the control group. -CHF patients had higher Serum NT-ProBNP level -Study conclude that patients with CHF, the MTP and ROS level of PBMCs are correlated with the changes in serum NT-ProBNP level	Song et al., 2016, Heart, Lung and circulation. [36]
Early stage HF patients *n* = 25, 12 male, 13 female Age: 49 ± 3 years EF: 67.40 ± 0.83 Control group *n* = 24, 11 male, 13 female Age: 47 ± 3 yearsEF: 69.63 ± 0.99	PBMCs sample	Mitochondrial respiration (Oroboros)	Measurement of inflammatory factors: High sensitivity C-reactive protein (hs-CRP), IL6, and TNF-⍺ -Oxidative stress biomarker: MDA Antioxidant system: SOD By using ELISA		Decreased mitochondrial oxygen consumption in HF compared to control group. -Inflammatory factors were significantly higher in patients with early stage HF. -SOD reduced, but MDA stayed unchanged in diseased patients.	Li et al., 2015 Scientific Report. [29]
HF patients with left ventricular assist device *n* = 10, 8 male, 2 female Age, median (range): 65 (57–69) EF% (median (range): 15 (10–20) Control group *n* = 10, 8 male, 2 female Age, median (range): 63 (26–74) EF %: NA	PBMCs (Circulating blood leukocyte)	N/A	-Detection of ROS in leukocyte by flow cytometry, and immunofluorescence microscopy -Antioxidant defense system; SOD in erythrocyte was measured by spectrophotometry. -oxidized low density (oxLDL) lipoproteins were analyzed in plasma, by ELISA. -DNA damage markers were assessed in blood lymphocyte, and measured by immunofluorescence microscopy	N/A	-In HF patients, the mean fluorescence intensity (MFI) of DCF-DA exhibited increased level of ROS in peripheral blood leukocyte than in control group. Post-operative value (1 week): Neutrophils ROS (+51%) Lymphocytes ROS (+37%) Monocytes ROS (+54%) -Quantity of ROS reach the highest 3 months later (value not specified) -SOD level decreased in HF patient than in control. And continue to decrease to reach the minimum at 3 months post-operative. -oxLDL were markedly higher in HF than in control group. These results suggested increased oxidative stress among HF patients which leads to mitochondria dysfunction. -Markers used to express DNA damage, reveals abnormal DNA repair.	Mondal et al., 2013, International Journal of Medical Sciences. [47]
Congestive heart patients (CHF) *n* = 15 9 Male, 6 female Age: 79 ± 9 EF% =37 ± 17 Control group *n* = 9 6 male, 3 female Age: 49 ± 22EF% =63 ± 5	WBC and Platelets blood sampling from radial artery, brachial vein and coronary sinus	N/A	Oxidative stress (immunofluorescence microscopy analysis of nitrotyrosine) -cytoplasmic oxidative stress (incubation of resuspended buffy coat with 5-6 CM-DCF). -7 CHF and 6 health individuals were evaluated for Mitochondrial oxidative stress, (Mitotracker red CM-H2 XRos M7513 Probe). -Both cytoplasmic and mitochondrial oxidative stress (live- cells fluorescence microscopy and FACS)	N/A	CHF exhibited increased protein nitosylation in arterial and venous WBC than control. -Cytoplasmic oxidative stress in CHF was increased in venous and arterially localized WBC and platelets. -For coronary sinus sampling, the number of ROS was higher than in venous (946 ± 475 vs. 659 ± 428 per 10,000 cells). -CHF patients had elevated mitochondrial ROS in WBC and platelets than healthy group. The number of ROS-positive venous WBC and platelets is (478 ± 261 per 10,000 cells vs. 162 ± 81 per 10,000 cells for control group). While, ROS-positive arterial WBC and platelets is 471 ± 211 per 10,000 cells vs. 85 ± 42 per 10,000 cells for healthy group. This increased number of circulating ROS suggesting increase oxidative stress in HF patients.	IJsselmuiden et al., 2008, (CardiovascularMedicine. [48]
Acute CHF Edematous *n* = 15 male 9 female 7 Age: 72.6 ± 3.7 EF% 36.2 ± 5.1 Non-edematous *n* = 15 male 10 female 5 Age: 78.5 ± 2.8 EF% 35.3 ± 2.7 Control group *n* = 20 male 18 female 2 Age: 68.5 ± 1.6	PBMCs (Peripheral blood leukocyte) 10 mL venous blood sample was collected, 5 mL was anticoagulated and assayed for fluorescence staining	Mitochondrial transmembrane potential (MTP) in leukocyte was analyzed by flow cytometry	Intracellular oxidants formation was examined by DCF for 20 min at 37°. -Fluorescence was Detected by flow cytometry -Analyzing plasma factors nitrogen metabolites. -Lipid peroxides including (MDA, HNE) -inflammatory factors: IL6, and TNF-⍺ using ELISA.	-Cell apoptosis was measured by tunnel assay	In CHF, MTP of PBMCs was markedly decreased, with the weakening in edematous HF patients more than in non-edematous specifically in lymphocyte. -Intracellular oxidants of PBMCs were increased, with the highest was in monocytes. -Edematous CHF had higher DCF fluorescence level than the other CHF group. -Apoptotic cells percentage was higher in polymorphonuclear leukocyte (PMN) than PBMCs. -edematous leukocyte presented with higher percentage of apoptosis than another CHF group. -plasma nitrogen level, lipid peroxide, and inflammatory factors was higher in CHF than control.	Kong et al., 2001, Journal of the American College of Cardiology. [37]

Yo: years old; LPS: Lipopolysaccharide; SOD: Superoxide Dismutase; GPx: Glutathione peroxidase.

**Table 2:** Mitochondrial DNA in peripheral circulating cells and cardiovascular disease.

Population Characteristics	Study Design	Mitochondrial Function/mtDNA Copy Number	Oxidative Stress	Cell Viability/Apoptosis	Results	Reference
Ischemic stroke patients Total *n* = 350 Age: 60.9 ± 9.1 Male *n* = 246 Female *n* = 104 Control group *N* = 350 Age:60.4 ± 9.1 Male *n* = 246 Female *n* = 104	mtDNA in Peripheral Blood Leukocyte	-mtDNA content (rt PCR) -The ratio of mtDNA to NuclearDNA is used to estimate the number of mtDNA per cell	-oxidized glutathione (GSSG), and reduced glutathione (GSH), (enzymatic (method) -8-hydroxy-2’-deoxyguanosine (biomarker of oxidative DNA damage, ELISA)	NA	mtDNA content in peripheral leukocyte for ischemic stroke patients was significantly lower than the control group. *P* < 0.0001 mtDNA content evaluated for 150 ischemic stroke patients = 0.90, while in 50 control individuals = 1.20 -The level of GSSG and 8-hydroxy-2’-deoxyguanosine were higher in patients with ischemic stroke than on the control group. GSSG Ischemic stroke = 1.83 Control = 0.79 8-hydroxy-2´-deoxyguanosine ischemic stroke = 6.33 Control = 4.87 These results exhibited that oxidative stress was higher in patients with ischemic stroke than in control group	Lien et al., 2017, Journal of American Heart Association [91]
3 cohort study with a risk factor of CVD 1st: Cardiovascular Health Study (CHS) *n* = 4830 Age: >65 years 2nd: Atherosclerosis Risk in Communities (ARIC) *n* = 11153 Age: Between 45 to 65 years 3rd: Multiethnic Study of Atherosclerosis (MESA) *n* = 5887 Age: 45 to 85 years Control: NA	In CHS: DNA was extracted from the buffy coat using salt precipitation following proteinase K digestion In ARIC: DNA was extracted from the buffy coat of whole blood using (Qiagen) In MESA: DNA was extracted from leukocyte using (Qiagen)	In ARIC and MESA, mtDNA copy number was measured by using prob intensities of mitochondrial single nucleotide polymorphisms (SNP) on the Affymetrix Genome-Wide Human SNP Array 6.0 IN CHS: mtDNA was calculated using multiplexed TaqMan-based PCR	NA	NA	-The effect of mtDNA copy number on the incidence of coronary heart disease was higher than in stroke and in other CVDs In all 3 cohort groups, the mtDNA copy number was inversely associated with CVD events	Ashar et al., 2017, JAMA Cardiology [79]
Coronary Heart Disease (CHD) classified in 4 groups according to Gensini score 1-Gensini score: 0-–22 *n* = 99, Male 72, Age: 57.3 2-Gensini score: 22–55 *n* = 98, Male 73, Age:57.9 3-Gensini score: 55–96 *n* = 102, Male 79, Age: 58.3 4-Gensini score:96–254 *n* = 101, Male 86, Age: 58.8 -Control group*n* = 110Age: 58.1	mtDNA of Leukocytes for CHF categorized by Gensini score	-genomic DNA was isolated from peripheral blood cells by E.Z.N.A blood DNA Midi Kit. -mtDNA quantification (Quantitative real time PCR).	NA	NA	mtDNA content of PBMCs was lower in CHD patients than in the control group. -mtDNA was reduced significantly, while Gensini score was increased suggesting the level of circulating mtDNA correlates with presence and severity of CHD.	Liu et al. 2017, Atherosclerosis [82]
Acute coronary syndrome (ACS) Total *n* = 14 Divided into 2 groups 1st group: (Survivor) who survive during 30 day of hospitalization *n* = 11, male 9, female 2 Age: 53 2nd group: (deceased) who died due to ACS during time of analysis *n* = 3 female Age: 87	Blood samples were collected from platelet poor plasma	-To evaluate mtDNA. Isolation performed with PROBA-NK reagent kit. -quantitation of mtDNA was performed by PCR	NA	NA	-Deceased group: the level of mtDNA level was higher (5900 copies/mL) than the survived group (36 copies/mL) *p* = 0.049 -increased level of mtDNA in plasma suggest a probability of death of 50% for ACS patients	Sudakov et al., 2017, European Journal of Medical Research [87]
Patients from the Atherosclerosis Risk in Communities (ARIC) *n* = 11093 male *n* = 4971 female *n* = 6122 Age: 57.9 ± 6.0	mtDNA in peripheral blood buffy coat	mtDNA copy number was measured by using prob intensities of mitochondrial single nucleotide polymorphisms (SNP) on the Affymetrix Genome-Wide Human SNP Array 6.0	NA		-Inverse association between mtDNA copy number and sudden cardiac death	Zhang et al., 2017, Eur Heart Journal [92]
Acute myocardial infarction patient undergoing primary angioplasty *n* = 55 male *n* = 47 female *n* = 8 Age: 57.4 ± 11.4 years Control group: *n* = 54 male *n* = 44 female *n* = 10 age: 55.3 ± 7.4	Peripheral blood leukocyte	Leukocyte mitochondrial DNA copy number (MCN) was measured from venous blood using PCR -AMI patients were divided into two groups according to median baseline leukocyte mtDNA copy number = 82/cell 1st group MCN ≥ 82 2nd group MCN < 82	NA	NA	-Baseline characteristics: In AMI patients the plasma leukocyte mtDNA copy number was significantly lower than in the control group. 122.7 ± 109.3 vs. 194.9 ± 119.5/cell *p* = 0.003 -AMI patients with lower MCN, had higher left ventricle shape sphericity index (SI), at 1,3,6 months after angioplasty and higher left ventricle diastolic and systolic volume at 6 months after angioplasty.	Huang et al., 2017, Circulatiog Journal, [90]
Patients with diabetes mellitus and atherosclerosis cardiovascular disease Total *n* = 275 -only Atherosclerosis: *N* = 55 Female 18 Age:60 ± 10 -only DM: *N* = 74 Female 47 Age: 55 ± 10 -Atherosclerosis and DM *N* = 48 Female 31 Age: 62 ± 8 Control group *n* = 98 Female 49 Age: 55 ± 7	PBMCs	Measuring mitochondrial DNA damage in PBMCs by PCR. -Total DNA was separated using QIAmp DNA mini kit and quantification determined by using Pico-green assay kit	Oxidative stress of arterial pulsatility (increased baseline pulse amplitude *p* = 0.009)	NA	Mitochondrial DNA damage was higher in all 3 diseased group, as compared with controls, with the highest in the group combining atherosclerosis and diabetes. -mtDNA measured in DM alone (0.65 ± 1.0) -mtDNA measured in atherosclerosis alone (0.55 ± 0.65) -mtDNA measured in both atherosclerosis and DM (0.89 ± 1.32) *p* < 0.05 mtDNA damage correlated with baseline pulse amplitude	Fetterman et al., 2016, (Cardiovascular Diabetology) [86]
General population Total *n* = 701 Divided by 3 tertiles of mtDNA content -Tertile 1 mtDNA content 0.39–0.86 *N* = 233 Female 103 Age: 51.6 ± 16.8 EF% 61.3 ± 7.0 -Tertile 2 mtDNA content 0.86–1.10 *N* = 234 Female126 Age:53.5 ± 14.7 EF%: 63.3 ± 6.56 -Tertile 3 mtDNA content 1.11–3.06 *N* = 234 Female 128 Age:54.3 ± 14.2 EF%: 62.9 ± 6.65	Peripheral blood cells	To assess the circulating mtDNA content, PCR was used. Total DNA was extracted from peripheral blood sample using QIAmp DNA Mini Kit.	NA	NA	There is a relation between peripheral blood mtDNA copy number and left ventricular function. Higher mtDNA content was associated with better systolic and diastolic left ventricular function	Knez et al. 2016, International Journal of Cardiology [77]
Chronic Heart Failure Total *N* = 1700 -Ischemic HF *N* = 790 Male 543 Age: 62.6 ± 10.4 EF% 57 -Nonischemic HF *N* = 910 Male 572 Age: 53.8 ± 14.3 EF% 40 Control group *n* = 1700 male 1115 Age: 57.7 ± 11.0 EF%: NA	Circulating Leukocyte -Blood sample were drawn, and leukocyte were isolated in K2-EDTA tubes.	Total DNA was extracted by using QG-Mini80 workflow with a DB-S kit. And DNAs of cardiac tissues were isolated by using QIAmp DNA Mini Kit. And copy number ratio was evaluated.	ROS were quantified in heart tissues using Dihydroethidium (DHE) staining. -In lymphocyte intracellular ROS was analyzed by flow cytometry using DCFH-DA -LDL was detected		HF patients presented a low mtDNA content compared to control group. Median 0.83, IQR: 0.60–1.16 vs. median 1.00, IQR: 0.47–2.20)*P* < 0.001. Ischemic HF patients are more susceptible to lower mt DNA copy number(Median 0.77, IQR: 0.56–1.08) than non-ischemic HF median 0.91, IQR 0.63–1.22 -mtDNA content of leukocyte was not correlated with LV diameter *p* = 0.988 -in HF group, LDL was associated with the mtDNA copy number *p* = 0.007 -Lower circulating mtDNA was correlated with increased risk of HF, *p* < 0.001 -In HF patients, the level of ROS was higher than in control group in heart tissues and in lymphocytes.	Huang et al., 2016, Medicine [89]
Coronary heart Disease Patients *N* = 378 Male 279 Female 99 Age: 57.9 -Control group *n* = 378 male 279 female 99 Age: 58.9	Peripheral Blood Leukocytes -5 mL of venous blood was drawn from each individual and anticoagulated into sodium citrate tube.	-DNA was separated from peripheral blood leukocyte using E.Z.N.A blood DNA Midi Kit. -DNA content was measured using PCR	NA	NA	-mtDNA content was inversely related to increased risk of CHD -CHD group shows marked lower mtDNA content, compared to controls, *p* < 0.001, -CHF had higher neutrophils counts compared to controls (5.10 ± 1.66 vs. 4.50 ± 1.51) but no difference in WBC count *p* = 0.154	Chen et al. 2014 (Atherosclerosis) [88]
Myocardial infarction ST segment elevation MI (STEMI) *n* = 20, 5 femaleStable angina pectoris (SAP) *n* = 10, 1 female Both undergoing percutaneous coronary intervention (PCI) and categorized as transmural or non-transmural Age: between 30 and 75 years	Platelet poor plasma	Venous blood sample were gathered, and DNA was extracted from platelet poor plasma using QIAmp DNA blood Mini Kit -Quantification of mtDNA using real time PCR	NA	NA	-Baseline characteristics: Both groups were similar except SAP group which received more PCI treatment than the other group. -After PCI: 3 h later, mtDNA plasma level of NADH dehydrogenase subunit 1 (ND1) were increased in STEMI compared to SAP. *p* = 0.01 -patients with transmural: ND1 levels were greater in STEMI patients *n* = 10, than STEMI patients with non-transmural *n* = 6 -positive correlation between the severity of myocardial damage and the level of mtDNA, mtDNA being increased in myocardial infarction.	Bliksøen et al., 2012, [83]
